# The Boss Is Back in Town: Insights into the Wolf Recolonization of a Highly Anthropized and Low-Ungulate-Density Environment

**DOI:** 10.3390/ani15131958

**Published:** 2025-07-03

**Authors:** Lorenzo Frangini, Giacomo Marzano, Alice Comuzzi, Andrea De Giovanni, Andrea Gallizia, Marcello Franchini, Michela Rugge, Marco De Luca, Giuseppe De Matteis, Stefano Filacorda

**Affiliations:** 1 Department of Agricultural, Food, Environmental and Animal Sciences, University of Udine, Via Sondrio 2/A, 33100 Udine, Italy; 2 Management Consortium of the Torre Guaceto Marine Protected Area and State Natural Reserve, Via Sant’Anna 6, Carovigno, 72012 Brindisi, Italy; 3 Independent Researcher, 73100 Lecce, Italy; 4 Biodiversity and Ecology Appennine Research Center, Contrada Palombarette 34, Pollenza, 62010 Macerata, Italy; 5 Department of Cultural Heritage, University of Salento, 73100 Lecce, Italy; 6 WWF Italia, Le Cesine State Natural Reserve, Masseria Cesine, Vernole, 73029 Lecce, Italy; 7 Coop Seges, Via 11 Febbraio 6, Vernole, 73029 Lecce, Italy

**Keywords:** *Canis lupus*, human-dominated landscape, activity pattern, livestock predation, pet predation, diet

## Abstract

This study documents the recolonization of the wolf using opportunistic and systematic data, including camera trapping and scat surveys in south-eastern Italy. Wolf distribution was mapped using a 10 × 10 km grid, and Generalized Linear Models (GLMs) were applied to identify environmental and anthropogenic drivers of stable presence. Camera trapping and scat analyses were used to provide the first insights into the activity patterns and feeding habits of the species. The results showed a significant expansion in wolf range, with occurrence increasing by 120%, especially in areas with high forest cover. During 1402 camera-trap nights, wolves and wild prey species displayed primarily nocturnal activity, reducing overlap with human presence, though prey activity peaks were offset from those of wolves. Diet analysis of 75 scats revealed a high occurrence of livestock and pets, along with all wild species living in the area (i.e., ungulates, medium-sized and micromammals). These findings underscore the wolf’s ecological adaptability and ability to thrive in heavily altered landscapes. Indeed, this study shows how wolves were able to occupy even suboptimal areas, taking advantage of both anthropogenic and wild trophic resources, still displaying nocturnal activity to lower the probability of encounters with humans.

## Simple Summary

After more than 100 years, grey wolves have returned to the Salento Peninsula in southern Italy—a region heavily shaped by human activity and with few wild prey species. This study followed the return of wolves in this area from 2014 to 2024 to assess their occupancy, behavior and diet. We observed that at first, (i) wolves settled near coastal areas, then gradually moved inland. By the end of this study, they had more than doubled the number of occupied areas compared to the earlier years. They preferred places with more patches of forest, which likely offered better cover and less disturbance. They were (ii) mostly active at night, as were other wild species, helping them avoid people. However, wolves and their prey tended to be active at slightly different times, possibly for the latter to reduce the chances of encounters with wolves. (iii) The analysis of wolf scats showed they often fed on farm animals and pets, but also on some wild animals. These results show how adaptable wolves are, even in places with lots of people and few wild species. This study highlights the importance of long-term monitoring and careful management to help people and wolves coexist.

## 1. Introduction

After centuries of persecution, large carnivores have faced a notable recovery in Europe, re-occupying many areas of their former ranges [1]. Large carnivores are defined as all carnivores weighing more than 15 kg [2], and they are key elements for ecosystems, providing top-down effects through the regulation of herbivore and smaller carnivore populations [2,3,4]. This effect can be exerted through consumptive effects (i.e., direct killing) [5], as well as non-consumptive effects, which are behavioral changes across the food web expressed as responses to predation risk [6,7]. Indeed, the latter has led to the definition of the ‘Landscape of fear’, which describes the spatial variation of fear as perceived by prey across their foraging or home range [6]. Despite the fact that the restoration of ecosystems induced by the large predators’ comeback is still debated [8], there is substantial evidence of their regulation of smaller predators [4,9,10], with positive effects on prey populations [11].

Regarding the grey wolf (*Canis lupus*) (hereafter, wolf), the key factors of such positive trend, which has promoted an almost doubling of its numbers within the last 10 years [12], are the conservation efforts promoted worldwide [13], along with the species’ ecological plasticity [12]. In Italy, the Apennine wolf (*C. l. italicus*) went nearly extinct, with approximately 100 individuals left in the central–southern Apennines in the 1970s [14]. After conservation policies, the wolf population expanded, reaching and recolonizing the Alps, with a population estimate of 3307 individuals (95% CI 2945–3608) in 2020–2021 over the entire Italian peninsula [15]. At the beginning of the recolonization process, the wolf settled mainly within forested areas with low human disturbance [16], leading the species to be considered highly sensitive to anthropic areas [17,18]. However, once these areas have been occupied, it is likely that dispersing wolves had to adapt to more fragmented and heavily anthropized areas [19,20,21]. Therefore, anthropogenic disturbance does not seem to be a limiting factor for wolf dispersal and settlement [21], with the species living wherever food is available and the human persecution is low [22]. In Italy, these areas are represented by hilly and lowland areas [19,20], and in most of them wolves can benefit from several and abundant trophic resources, such as wild and domestic prey [23,24].

Within southern Italy, the area mostly occupied is represented by the central–western side (i.e., the administrative regions of Calabria and Basilicata), where the Apennine chain lies, with highly spread forest cover and ungulate presence [25,26]. However, the eastern side is represented mainly by the Apulia region, where, in 2016, the wolf presence was reported in the central and northern areas [25,27]. These areas are represented by the border with Basilicata in the west, and the Gargano National Park in the east [25,27], both in mountainous and hilly landscapes. However, most of the Apulia region is a flat area covered by agricultural lands and urban areas, covering the 80% of the regional surface, with natural forested areas covering less than 10% [28]. The southernmost area of Apulia region displays the highest anthropized levels, corresponding to the provinces of Lecce, Taranto and Brindisi (hereafter, Salento), with over 90% of patching consisting of agricultural and urban areas [28]. Despite the fact that Salento does not seem to represent the most suitable area for the wolf, in 2017, Marzano et al. [29] reported the first confirmed reproduction after a century of disappearance. Before their sightings, the latest records of the wolf dated back to the 19th century [30], when its main prey species were already extinct (i.e., red deer *Cervus elaphus*, the roe deer *Capreolus capreolus* and the wild boar *Sus scrofa*) [31].

Nowadays, in Salento, large herbivores are still absent [32], with only a small nucleus of hybrid wild boars, located in a protected area, and few dispersing individuals that were illegally introduced in the 2010s (Marzano pers. comm.). The Salento Peninsula therefore represents one of the few areas in Italy where the wolf’s main prey are absent. Within this context, wolves can adapt their food habits to other food categories and also use anthropogenic food, such as domestic ungulates, poultry [33], and even smaller mammals [34]. Here, the main medium-sized mammals are represented by European hare (*Lepus europaeus*), the red fox (*Vulpes vulpes*), the European badger (*Meles meles*) and the beech marten (*Martes foina*) [32]. Therefore, the recolonization of Salento represents a unique scenario in Europe, where the wolf might have adapted to a highly anthropized environment, providing further insights on the species’ ecological plasticity.

The main aim of our study was to update the current knowledge on the wolf distribution in the Salento Peninsula, providing an overview on the recolonization dynamics over eleven years of data collection (2014–2024). Specifically, we aimed to (i) map the wolf distribution range along the years and the evolution of the reproductive events, and to (ii) assess ecological and human-related factors potentially explaining the observed patterns. Moreover, we used data collected from systematic surveys to preliminary assess (iii) the activity pattern and temporal overlap of humans, the wolf and its potential prey, as well as (iv) to characterize the main food items within its diet.

## 2. Materials and Methods

### 2.1. Study Area

**Figure 1:**
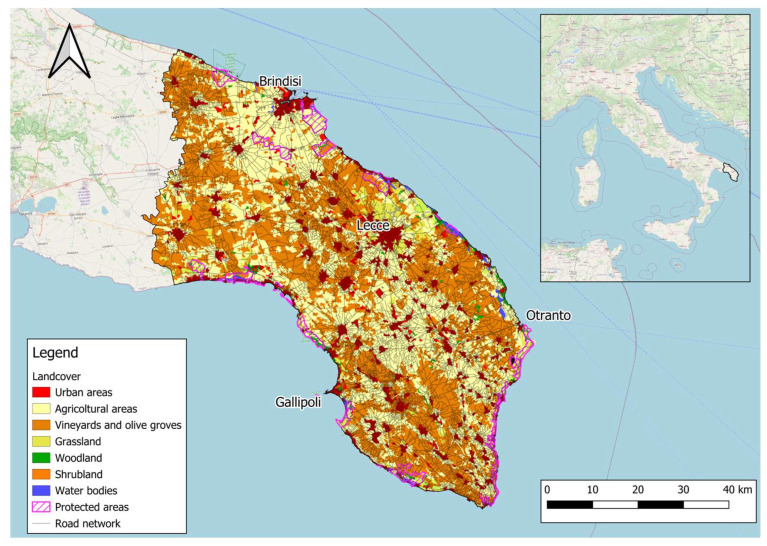
Study area with the main landcover types derived from Copernicus Corine Land Cover. Most of the surface is covered by agricultural fields, vineyards and olive groves. Areas depicted in purple represent protected Natura 2000 sites on land (marine protected perimeters are not depicted). The highly dense road network is shown with black lines and is denser in the southern district of the study area. Background represented by the OSM Standard (Sources: © Open Street Map).

Our study area corresponds to the Salento Peninsula, located in the Apulia region. It encompasses the administrative province of Lecce and, partially, those of Brindisi and Taranto, covering an area of 4076 km^2^ (Figure 1). This area represents the south-easternmost part of Italy, stretching for 120 km between the Ionian and Adriatic Seas as a flat area with most of the landscape below 100 m a.s.l. The climatic characteristics are Mediterranean, with rainfalls during fall and winter and long sunny and dried periods in summer. Here, the temperatures are mitigated by the presence of the sea, and winds are highly variable in intensity and direction [35]. From a vegetational point of view, the Salento Peninsula is the meeting point between the Eastern Mediterranean Basin flora and that of the rest of Italy [36]. Little is known about the terrestrial mammal community in this area, but the main medium-sized mammals are the European hare, the red fox, the European badger and the beech marten. The human-dominated landscape is characterized by urban (8.76%) and agricultural patches (mostly agricultural lands 31.5%, olive groves 42.79% and vineyards 7.83%), with few and highly fragmented natural areas (6.35%), mostly spread along the coastline, where the protected areas are located [28]. Moreover, the province of Lecce has the highest density of municipalities in Italy, resulting in a very dense road network, with an average of 4.31 km/km^2^.

### 2.2. Data Collection and Processing

We investigated the presence of the wolf both opportunistically (2014–2024) and systematically through camera trapping and scat collection (2023–2024). Opportunistic data spread over different data sources: carcass occurrences (mainly road-killed individuals), predation on domestic animals (pet and livestock), camera-trap images and direct observations recorded and verified by experts. We kept only independent events to avoid biases in the maps (see Section 2.3), retaining only one occurrence for each day within the same area.

From January 2023 to December 2024, we monitored wolf presence using 17 camera traps deployed within three monitoring sites (Site 1 = 11 cameras, Site 2 = 4 cameras, Site 3 = 2 cameras; average distance between sites 51.2 km), strategically deployed along trails and forestry roads. We chose these sites based on previously collected data, i.e., where the reproduction was confirmed in the past years, and the presence of the species was stable along the study period. Cameras were set to acquire videos of 20 s and were inspected monthly to download data and replace batteries. Data were classified using Timelapse Image Analysis system software (version 2.3.3.0) [37], and for the activity pattern and overlap analyses, we kept only independent detections for each species, discarding all images of the same species within a 30 min interval [38]. Since some of the cameras were placed close to others (e.g., <250 m), and these monitoring sites might display high detection rates due to the pooled wolf activity, we kept independent detections, considering the monitoring sites instead of single cameras to avoid biases in the analyses.

We collected wolf scats along 19 linear transects that we inspected monthly in June–December 2024. On average, transect length was 957 m (min = 346 m, max = 1609 m), and they were placed along hiking trails and forestry roads previously identified based on the knowledge of the species’ presence or known marking sites, trying to cover different areas and far from urban areas where domestic or stray dogs might have occurred. To avoid misidentification with non-wolf scats (fox and domestic dog), since we did not use genetic analyses, we considered morphology (i.e., shape, color, mass, preliminary content and a minimum diameter of >30 mm) and the location where each scat was deposited [39]. Scats were stored at −20 °C until the analysis, and before the processing they were sterilized at 90 °C for 6 h. Following Lovari et al. [40], fine sieves with 0.5 mm meshes were used to wash scats and separate hair from bones, feathers and other material. Hairs (cortical scales, medulla and root characteristics) of mammals and bones were microscopically identified with the aid of literature references [41,42] and specimens from local mammal species. Food items were grouped in five categories: livestock, wild prey, pet, fruit and other. We defined the seasons based on the date of collection: spring (20 March–21 June), summer (21 June–22 September), fall (22 September–21 December) and winter (21 December–20 March).

All opportunistic and systematic data were classified following the SCALP (Status and Conservation of the Alpine Lynx Population) criteria [43], to ascertain the correct species identification and assess the data reliability [44]. Therefore, data were classified into three categories: C1, representing “hard evidence”; C2, “confirmed observations”, validated by experts; C3, “unconfirmed observations”, not validated [44,45]. As suggested by Marucco et al. [46], we removed all the C3 occurrences to avoid biases in the analyses.

### 2.3. Data Analyses

#### 2.3.1. Wolf Distribution

To display the recolonization dynamics of the wolf, we used a 10 × 10 km grid to map its presence during 2015–2024. We chose this period because in 2014 we obtained only C3 data, and we adapted the guidelines suggested by the Wolf Alpine Group [47] and Ranc et al. [48]. We split this period into two equal time intervals (i.e., 2015–2019 and 2020–2024) to better appreciate the recolonization process. Similar to Ranc et al. [48], we used C1 and C2 occurrences to define wolf presence as ‘Permanent’ or ‘Sporadic’. A grid cell was defined as ‘Permanent’ when, in at least in half of the period (i.e., 3 years), one C1 or two C2 occurrences were detected per year, or a reproduction event was detected in the last three years. Similarly, a grid cell was defined as ‘Sporadic’ when it contained one C1 or C2 in less than half of the period (i.e., 3 years) of each interval. Species absence was assessed when no data were recorded for the species, neither systematically nor opportunistically. Independent reproduction events were defined when pups were recorded simultaneously within locations at least 20 km away, to be conservative in a more homogenous landscape such as Salento [49].

To test for environmental covariates likely explaining the distribution status of the wolf at a broad scale, i.e., permanent (1) vs. sporadic (0), we performed a Binomial Generalized Linear Model (GLM), that is, a logistic regression, using each grid square as a sampling unit. We investigated only grid squares within the province of Lecce, since some of the covariates were available only for that province. We chose a set of landscape covariates representing both human footprint (road density, the number of ovicaprine farms per square grid) and environmental conditions (percentage of protected area and the percentage, total area and number of patches of vegetational cover). We defined as vegetated areas all shrub and woodland patches that we derived from Lavarra et al. [28]. Human footprint covariates were chosen as surrogates for anthropogenic food resources (i.e., scavenging on road-killed animals [50] and preying on livestock [51,52]), while environmental covariates represented both shelter and trophic resources. We tested for multicollinearity through the Pearson’s correlation coefficient (r), removing covariates with r values greater than 0.7. Moreover, we assessed the model assumptions using ‘DHARMa’ R package (version 0.4.7.) [53]. We tested for several model combinations, and model ranking was performed based on the AIC [54] and ΔAIC [55]. In the presence of models showing ΔAIC < 2 (hence, considered competitors of the best model; [56]), we performed model averaging by calculating the Akaike’s weight (ωi), which expresses the relative amount of variation explained by each model compared with all other models [55]. Model fitting and selection were performed through ‘MuMIn’ R package (version 1.48.11.) [57].

Moreover, when a new reproductive event occurred, we reported grid square environmental characteristics to highlight if a gradual shift toward suboptimal areas occurred.

#### 2.3.2. Activity Pattern and Overlap

To assess the wolf activity patterns, we estimated species-specific activity patterns and temporal overlap via non-parametric kernel density estimation [58]. To quantify temporal overlap between pairs of species, we used the Δ coefficient of overlapping and, referring to Meredith & Ridout’s method, Δ4 (Dhat4) was used for large sample sizes (number of independent events > 75). Overlap coefficients were classified as ‘low’ (Δ < 0.50th percentile), ‘moderate’ (0.50th ≤ Δ ≤ 0.75th percentiles) or ‘high’ (Δ > 0.75th percentile), as proposed by Monterroso el al. [59]. All calculations were performed using the ‘overlap’ R package (version 0.3.9.) [60].

#### 2.3.3. Diet Analysis

We assessed the frequency of occurrence of both food categories and each item in the wolf diet by analyzing wolf food habits through the identification of indigested remains in scats. For each food category, we calculated its frequency of occurrence in the diet as the percentage of scats containing it. Then, we calculated bootstrap 0.95 confidence intervals of frequency of occurrence through 1000 replicates [38]. We calculated the Brillouin index [61] and its incremental change, and we assessed the minimum number of scats necessary to study the wolf diet when the incremental change declined below 1% [62]. Calculations were performed using the ‘tabula’ R package (version 3.3.1.) [63].

## 3. Results

### 3.1. Wolf Distribution

**Figure 2:**
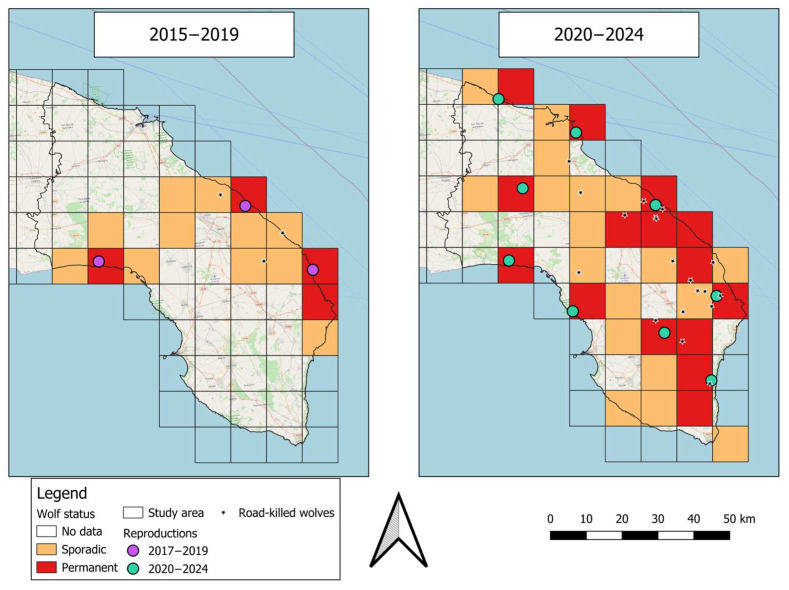
Species’ distribution in the two study periods as a 10 × 10 km square grid system. Distribution maps were generated using C1 and C2 data. For each period, the locations of road-killed wolves (black stars) and reproduction sites (colored points) are represented. Background represented by the OSM Standard (Sources: © Open Street Map).

Overall, we obtained 816 (opportunistic = 176; camera trapping = 565; scat sampling = 75) wolf occurrences, which were reduced to 454 independent events (Table 1). The overall number of occurrences increased significantly in 2020–2024 (Table 1): both the number of road-killed wolves (2015–2019 = 3; 2020–2024 = 19) and the reproduction events (2015–2019 = 3; 2020–2024 = 8; Figure 2) increased in the second period. Consequently, the number of occupied square grids increased in the second period (*n* = 18, a net increase of 120%), especially for the ‘Permanent’ square grids, with a net increase of 275% (*n* = 11) (Table 1 and Figure 2). The areas mostly occupied (i.e., ‘Permanent’ status) in the first period were located along the eastern and western coastlines, while in the second period they spread both along the coastline and toward the inner areas of the peninsula, with new confirmed reproduction sites (Figure 2). During the study period, square grids that experienced new reproductions were characterized by a lower percentage of forested areas and an increasing number of ovicaprine farms (Table 2).

**Figure 3:**
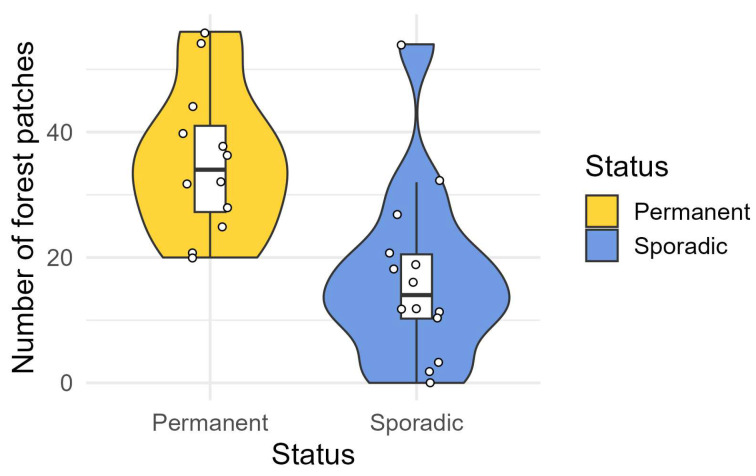
Violin (colored) and box plots (white) showing the number of forest patches in ‘Permanent’ and ‘Sporadic’ square grids in the 2020–2024 period. Scattered dots represent raw data, i.e., the number of forested patches in each square grid (*n* = 26).

When testing the wolf status (*n* = 26), we removed the total area of vegetated patches due to multicollinearity issues. We performed 32 different models: the best was represented by the one with only the number of forest patches (Table 3), where its value was statistically greater in the ‘Permanent’ square grids than in the ‘Sporadic’ ones (Figure 3).

### 3.2. Activity Pattern and Overlap

**Figure 4:**
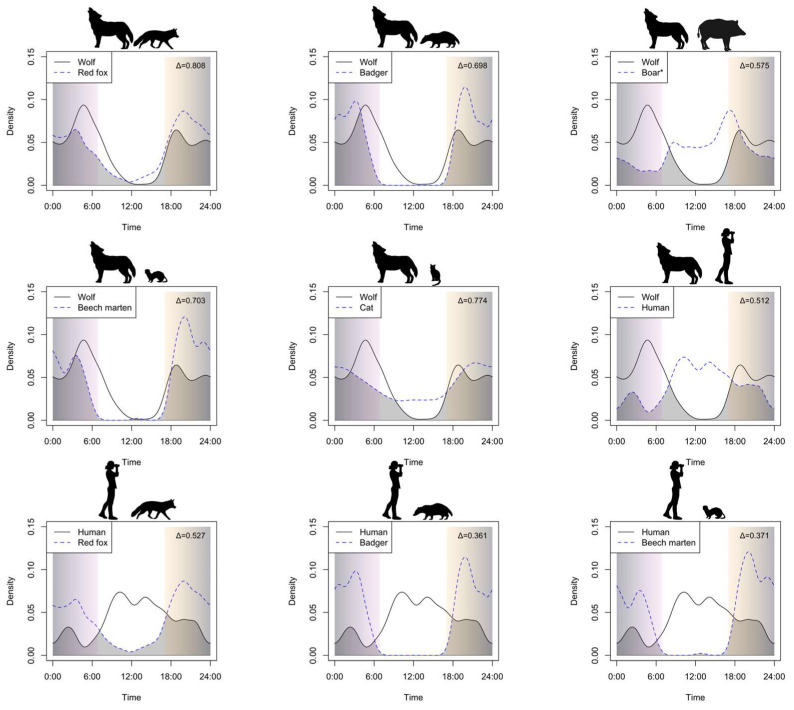
Activity pattern and overlap between wolf and its potential prey, as well as humans and wild species. The grey-colored area represents the overlap area whose value is depicted in the top-right side of each panel. The vertical shadow areas represent sunrise (on the left, pink-colored) and sunset (on the right, beige colored) in each panel. From top-left to bottom-right, each panel represents the overlap between wolf–red fox, wolf–badger, wolf–wild boar, wolf–beech marten, wolf–domestic cat, wolf–humans, human–red fox, human–badger and human–beech marten. ‘*’ in boar indicates that the species is not properly wild, due to hybridization with domestic pigs.

Overall, we monitored the three camera trapping sites for 1402 camera-trap nights. We detected 408 independent events for wolves and 867 for people. Among the potential prey, the highest detections were displayed by the red fox (*n* = 1845), followed by the badger (*n* = 339), the wild boar (*n* = 303), the beech marten (*n* = 222) and the domestic cat (*n* = 79) (Table 4). Wild boars were exclusively documented in one of the three sites. Across the study area, wild species (i.e., the wolf, the red fox, the badger and the beech marten) exhibited crepuscular and nocturnal activity, the domestic cat displayed a slight nocturnal activity, while people and wild boar were mostly diurnal (Figure 4). Wolves showed high temporal overlap with the red fox (Δ4 = 0.808) and the domestic cat (Δ4 = 0.774) and moderate overlap with the beech marten (Δ4 = 0.703), the badger (Δ4 = 0.698), the wild boar (Δ4 = 0.575) and human activity (Δ4 = 0.512) (Figure 4). On the other hand, people exhibited a moderate temporal overlap with the red fox (Δ4 = 0.527) and low overlap with the beech marten (Δ4 = 0.371) and the badger (Δ4 = 0.361) (Figure 4).

### 3.3. Wolf Diet

**Figure 5:**
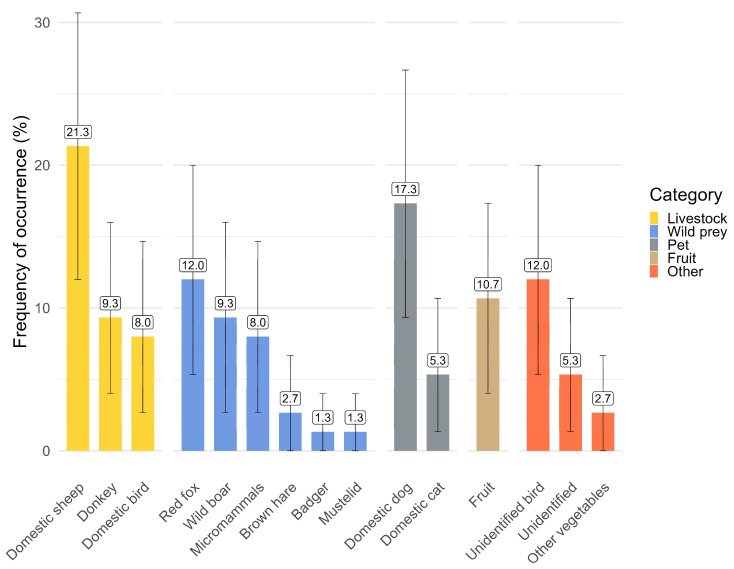
Food habits of the wolf (June–December 2024, *n* = 75 scats): percentage of different food categories in the diet, expressed as absolute frequency of occurrence. Error bars indicate 0.95 bootstrapped confidence intervals estimated through 1000 replicates. The color of the bars represents the macro category of each food item. Wild boars were grouped as wild prey, despite the fact that they cannot be considered wild species at all. The numbers at the top of each bar represent the absolute value of each food item.

We monthly covered 18.18 km and an overall 127.22 km looking for wolf scats. We collected 75 scats, mainly in summer (*n* = 33) and fall (*n* = 27), fewer in winter (*n* = 13), and only a few samples in spring (*n* = 2). Following the incremental change of the Brillouin index, the minimum number of scats required to study the wolf diet is 30. The two main food categories were represented by livestock (*n* = 28, 37.3%) and wild prey (*n* = 25, 33.3%), followed by pet animals (*n* = 16, 21.3%). Domestic sheep (*n* = 16, 21.3%) and dogs (*n* = 13, 17.3%) were the most common food items, followed by the red fox (n = 9, 12%) and unidentified remains of birds (*n* = 9, 12%) (Figure 5).

## 4. Discussion

The recolonization of the Italian Peninsula by the wolf started in the 1970s, and it occurred mainly along the forested areas of the Apennines and the Alps [64,65]. Despite its relative proximity to remnant nuclei in central-southern Apennines [65], the Salento Peninsula experienced the return of the wolf only in the last decade [29]. Using data collected over eleven years, we were able to update the current knowledge on the presence of this species in Salento, as well as to reconstruct the recolonization dynamics. Moreover, through data collected systematically in the last year (2023–2024), we preliminary assessed the nocturnal activity pattern of the wolves and their potential wild prey, with high and moderate temporal overlap among them. As a last step, the first insights provided by the scat analysis, revealed that domestic animals, both livestock and pet, are an important part of the wolf diet in our study area, along with the wild prey, mainly represented by the red fox.

At the beginning of their recolonization process (2015–2019), the wolves started to settle only along the coastline facing both the Adriatic and Ionian seas. In the second period, the wolf’s range exhibited an omnidirectional expansion, also occupying inner areas of the peninsula, although with a low probability of presence [26], with eight reproduction events occurring in the last year. Wolf dispersal and movement patterns have been widely studied, e.g., [66,67,68,69,70], but few studies have focused on a highly anthropized landscape [66,70]. As a general rule of thumb, offspring or even adult/old individuals disperse away from their natal pack to find new breeding territory [67,71]. Several factors can affect the dispersal pattern, such as habitat barriers, prey abundance, environmental configuration and even individual characteristics [67]. Moreover, within a highly fragmented and anthropized landscape, there is evidence that there may not be a uniform strategy to cope with disturbances, with some individuals approaching human settlements and others avoiding them [72]. One of the few factors that is in agreement across studies is the crucial role of canopy cover as refuge areas both for dispersal and persistence within these anthropogenic contexts [21,70,72]. Our results are in line with these findings since most of the vegetated patches lie along the coastline, along with the protected areas. It is likely that during the first period of recolonization of Salento, the wolf settled within the most suitable areas (i.e., with lower human disturbance) with the greatest number of vegetated patches. Here, it might have found undisturbed sites in which to reproduce, providing new dispersing individuals. In fact, once settled in a reproductive area, reproduction was confirmed in the successive years in all sites, with only one exception, similar to other anthropized scenarios [19]. In the second period, there might have been a saturation of the largest forested patches, leading therefore to the colonization of even suboptimal areas [16,19] in the inner part of the peninsula, where road network is particularly dense and forest cover decreases. Fardone et al. [21] suggested that where infrastructures are pervasive, wolves cannot avoid them, since this would mean disregarding large areas of available land. As a consequence, in our study area, the number of road-killed wolves increased significantly in the second period, being mostly located within the inner areas. Here, the presence of smaller and more fragmented cover areas may have led to greater displacements, forcing the individuals to cross risky roads and increasing the chances of being road-killed [73].

Camera-trap monitoring provided the first overview of activity pattern and overlap of the wolf and other wild species, and how they cope with human activity. All wild species, namely the wolf, the red fox, the badger and the beech marten, exhibited crepuscular and nocturnal activity, while the feral domestic cat showed less differentiation between day and night and, in contrast to other studies [74,75,76,77], the wild boar exhibited diurnal activity patterns. The observed patterns, and the avoidance or moderate temporal overlap between wildlife species and humans have been described worldwide [78,79]. Due to the strong human footprint of our study area, our results best reflect the patterns observed in other environmental contexts [76,79], with wild species being nocturnal to avoid the hours when human activity was higher. However, the wolves displayed a not negligible, but decreasing, activity after sunrise [80], which may be linked to the return to the rendezvous sites after nocturnal patrolling of their territory. Despite the general avoidance of human activity, the wolves and the red foxes exhibited moderate overlap with humans, likely due to a moderate activity level after the sunset. Since cameras were located in touristic protected areas and within the ‘Torre Veneri’ military training area (MTA), evening human activity may be linked to tourism or military trainings, respectively, therefore influencing the overlap levels. As it has been observed across Europe [81,82], MTAs provide crucial refugia in highly anthropized landscapes. Indeed, in the ‘Torre Veneri’ MTA, we reported one the first packs in Salento, reproducing every year since their establishment, despite military training activities, which did not negatively influence its presence. Across the three sites, human activity forced wild species to share the night hours, partially explaining the moderate/high Δ4 values among wild species, increasing the probability of interspecific interactions [83]. However, it is noteworthy that all the potential wild prey displayed activity peaks complementary to those of the wolves. Since we located camera traps in areas where the wolf presence has been detected for at least 2 years, we speculate that these activities may be a behavioral adaptation to cope with both the presence of an apex predator and that of humans [7]. On the other hand, the high overlap between the wolf and feral domestic cats, and the moderate overlap with the hybrid wild boars, may be linked to the lower avoidance of humans by these two species. Specifically, domestic cats did not show strong differentiation between day and night, with relatively higher activity levels during the day and lower during the night compared to wild species. Therefore, this is the reason why the wolf and domestic cat highly overlapped, making the latter a potential and effective prey (see below). The wild boars, as previously explained, were illegally introduced and hybridized with domestic pigs, likely being less fearful of humans and displaying daily peaks. Nevertheless, the overlap with the wolf was not particularly low, especially during the sunset, likely as a result of the modulation of wolf activity toward the boar as predatory strategy.

As a first overview on the diet in the Salento Peninsula, where the main prey are missing (i.e., large wild ungulates [84]), the wolves showed an opportunistic feeding behavior, feeding on a wide range of food items. In this area, domestic animals were the main dietary component, with high frequency of occurrence especially for sheep and domestic dogs. When large wild ungulates are absent or scarce, it has been shown that the wolf can adapt its diet to other food sources, such as domestic animals [51,52], especially when they are easy to prey on (e.g., livestock not protected by guardian dogs) [51,85]. Moreover, when living in highly anthropic areas, even if the wild prey abundance is high, livestock can be an important trophic resource, as observed in a hilly landscape of Central Italy [86]. In our study area, most of the livestock in Salento are represented by sheep kept within walled-up farms called ‘Masserie’, which were used in the past to protect livestock by bandits and predators [87]. However, in recent times, due to the local extinction of the wolf, sheep are let to graze in the pastures outside the walls, without proper mitigation measures, representing an easy prey. Moreover, square grids where the latest reproductions occurred were characterized by a higher number of ovicaprine farms, possibly suggesting that farms lie within the core areas of wolf packs [19]. However, it is noteworthy that we did not find any statistical relationship between the number of ovicaprine farms and the distribution status of the wolf. Therefore, despite the relative high frequency of domestic sheep within its diet, the wolf might not rely on the presence of farms to settle permanently within human-dominated landscapes when more natural areas are available. It is particularly interesting the relatively high frequency of domestic dogs in the wolf’s diet, which we speculate may be linked to the availability of stray dogs in the area (see [88]), along with pet dogs, which we reported as preyed on within private gardens. However, we did not detect stray dogs with camera-trap monitoring. Furthermore, during our study period, they apparently decreased in numbers, being spotted only nearby urban areas (Marzano pers. comm.). Such observations suggest that wolves may have negatively influenced the abundance and distribution of stray dogs, highlighting the need to investigate this topic. Among wild prey, the red fox was the most frequent item [52], which may be linked to its relative abundance in the area [89], as also detected by our camera traps. This has also led to lethal management control measures to reduce the species’ density. Within this context, future studies should focus on the possible top-down regulation on the red fox by the wolf, within packs territories, as it has been observed in Scandinavia [10]. Despite the fact that the results on the red fox contrasted with those found in other Italian areas, where it showed lower frequencies or was even absent [38,86], for other medium-sized mammals, we found similar frequencies, suggesting that mustelids may have a marginal role in the wolf diet [38,86]. However, scat analysis could not enable us to assess whether, and to what extent, wild and domestic animals were preyed upon or scavenged [33]. Indeed, we speculate that it is likely that some of the food items found within scats may have been scavenged along roads after being road-killed, as hypothesized for other wild canids [50], representing in turn a threat for wolves. Nevertheless, we urge researchers to consider our results as preliminary due to some potential limitations: lack of genetic analyses, small sample size, unbalanced seasonal collection and short transect length [19].

## 5. Conclusions

In this study, we present a first overview on the recolonization of the wolf in one of the relatively less suitable landscapes in Italy, in terms of low forest cover and ungulate presence, as well as high human pressure. Using opportunistic data collected over 11 years, we were able to represent the colonization dynamics of the peninsula: first, the wolf occupied the most suitable areas, represented by forest patches along the coastline where most of the protected areas are located, and through a saturation process, the species started to colonize even inner and more fragmented areas. Combining camera trapping and diet analysis, we provided the first insights into the ecological choices of the species in such an anthropized environment. We highlighted its well-known ecological plasticity, which enables it to exploit areas heavily used by humans by modulating its activity patterns during night hours to avoid direct encounters, while at the same time exploiting anthropogenic food resources. However, we stress that our results provide just a preliminary survey, with some limitations: opportunistic data provide crucial information, especially where no systematic surveys have been carried out, but might be geographically biased. Moreover, we performed camera trapping in only three areas, with different sampling efforts for each area. Finally, we did not collect the same number of scats for each season, and this did not allow us to evaluate seasonal variations in the diet. Despite such limitations, we provided insights into the high ecological plasticity of the wolf within a human-dominated landscape. We urge future studies to perform systematic surveys with different techniques to assess both the ecological relationships within the mammal community and the degree of human–wolf conflicts to best outline management strategies for wildlife conservation and promote human–wildlife coexistence.

## Figures and Tables

**Table 1:** Synthesis of data used to develop distribution maps displayed in Figure 2. We refer to unique reproductions as the maximum number of reproduction events that occurred in each period.

		2015–2019	2020–2024
Occurrence data with SCALP code	C1	43	377
	C2	4	30
	C3	/	/
	Tot	47	407
Number of square grids	Sporadic	11	18
	Permanent	4	15
	Tot	15	33
Number of unique reproductions		3	8

**Table 2:** Square grid characteristics during successive reproductions. Each row represents the square grid where a new reproduction occurred, with its related environmental characteristics.

Year of Reproduction	% Forest	Number of Forested Patches	Total Forested Area (km^2^)	Number of Ovicaprine Farms	% Protected Areas	Road Density (km/km^2^)
2017	36.73	28	11.61	6	0.72	4.55
2018	27.40	10	5.84	3	0	4.31
2019	27.37	32	7.98	2	0	3.17
2020	4.311	25	4.16	14	5.37	4.58
2020	4.09	40	2.92	12	8.30	4.14
2022	2.11	32	2.11	10	0	3.27
2023	2.2	22	0.96	/	5.89	4.55
2024	0.99	20	0.99	36	0.29	5.88
2024	0.70	7	7	/	0	1.36

**Table 3:** Ranking of Generalized Linear Models (GLMs), with the best model in shown in *italics*. The variables statistically significant are denoted by ‘*’.

Model	df	logLik	AICc	Δ AIC	Weight
Forest_count *	2	−12.5049	29.5316	0	0.2020
Forest_count * + PA_perc	3	−11.7519	30.5948	1.0632	0.1187
Forest_count * + Forest_perc	3	−11.7657	30.6224	1.0908	0.1171
Forest_count * + N_farms + Forest_perc	4	−10.3976	30.7000	1.1684	0.1126
Forest_count * + N_farms	3	−12.1603	31.4114	1.8798	0.0789

AIC, Akaike’s information criterion; df, degrees of freedom; logLik, log-likelihood; Δ AIC, the difference from the AIC value of the best model; Forest_count, number of forest patches; PA_perc, percentage of protected areas; Forest_perc, percentage of forested patches; N_farms, number or ovicaprine farms.

**Table 4:** Camera-trap data within three rendezvous sites. RAI, Relative Abundance Index, expressed as 100 × ((number of detections for each species)/(monitoring period)).

Monitoring Area	Number of Camera Traps	Camera-Trap Nights	Species	Number of Independent Detections	RAI
Site 1	11	732	Wolf	363	49.59
			Red fox	1548	211.48
			Badger	300	40.98
			Beech marten	215	29.37
			Wild boar	303	41.39
			Domestic cat	68	9.29
			Human	554	75.68
Site 2	4	480	Wolf	19	3.96
			Red fox	163	33.96
			Badger	28	5.83
			Beech marten	7	1.46
			Wild boar	/	/
			Domestic cat	8	1.67
			Human	221	46.04
Site 3	2	190	Wolf	26	13.68
			Red fox	134	70.53
			Badger	11	5.79
			Beech marten	/	/
			Wild boar	/	/
			Domestic cat	3	1.58
			Human	92	48.42

## Data Availability

The data presented in this study are available on request from the corresponding author due to sensitivity of the species’ reproduction sites.
